# Older adults’ perceptions of navigating eye health care in Denmark: a qualitative study

**DOI:** 10.3399/BJGPO.2023.0118

**Published:** 2024-03-20

**Authors:** Catharina Thiel Sandholdt, Marie Honoré Jacobsen, Olivia Hjulsager Mathiesen, Alexandra Brandt Ryborg Jønsson, Andrea Nedergaard Jensen, Emma Katrine Frøhlke Steinbo, Susanne Reventlow, Frans Boch Waldorff

**Affiliations:** 1 Research Unit for General Practice & Section of General Practice, Department of Public Health, University of Copenhagen, Copenhagen, Denmark; 2 Department of People and Technology, Roskilde University, Roskilde, Denmark; 3 Department of Public Health, University of Copenhagen, Copenhagen, Denmark; 4 Department of Ophthalmology, Zealand University Hospital, Roskilde, Denmark

**Keywords:** eye problems, eye, care of older people, aged, qualitative research

## Abstract

**Background:**

Vision impairment can have an impact on cognition, health, and social function. Vision loss may be avoided if detected early and treated promptly. Eye health is a minor topic in general practice, but the ongoing relationship between doctor and patient has the potential to assist the patient in navigating the healthcare system and guaranteeing timely healthcare service delivery.

**Aim:**

To explore the attitudes of older members of the public (aged ≥60 years) towards navigating primary sector eye health care in Denmark, with a focus on optometrists, practising ophthalmologists (POs), and GPs.

**Design & setting:**

Qualitative study in Copenhagen, Denmark.

**Method:**

Focus group interviews were performed in the spring of 2022 with 21 older members of the public.

**Results:**

Older members of the public perceived optometrists and POs to be the most relevant health professionals to consult about eye health. Opportunities were identified for enhancing the function of general practice including detecting early signs of visual impairment, being in charge of further referrals, and managing issues affecting quality of life such as dry eyes.

**Conclusion:**

Older members of the public sought help from health professionals who are directly qualified to treat symptoms of vision impairment that patients are experiencing or expect to face in the near future. Participants identified a potential for GPs to address vision impairment. This included focusing on the patient’s general health and function, as well as potential comorbidities influencing treatment trajectories. The current denigration of general practice risks missing out on the potential benefits of robust engagement from general practice in eye health.

## How this fits in

In general practice, eye health is a poorly investigated topic. This study has shown how older members of the public navigate their health-seeking behaviours in primary care for vision and eye health, including their impressions of optometrists, practising ophthalmologists (POs), and GPs. The study has shown general practice is not the first option for patients and expands on the facilitators for and barriers to engaging GPs in vision and eye health. The study has added to the discussion of how to design healthcare services.

## Introduction

Vision impairment is a common anxiety of function loss in older adults and can lead to worsened mental health.^1,2^ It may also have an impact on cognition and social function.^3–8^

Vision impairment can be caused by refractive anomalies, which can be corrected, or by ocular disease, which in many situations can be treated. This study took place in Denmark, where the majority of the population has access to corrective lenses and to healthcare services for the detection and treatment of eye problems.. As is the availability of healthcare services for the detection and treatment of eye problems. Owing to demographic changes the prevalence of eye problems, such as age-related macular degeneration (AMD), is expected to increase.^9^ A recent study found that anti-vascular endothelial growth factor (VEGF) medication reduced the prevalence of central vision loss in the Danish population,^10^ but issues with detection, especially of AMD and glaucoma, remains.^11–14^ Early detection and access to treatment are vital to slow the disease progression.

General practice is the first point of contact with the healthcare system, so it plays an important role in detection of vision impairment. General practice takes a person-centred approach to treatment and follows patients over time.^15^ Many patients who present to general practice have chronic conditions or are older. The ongoing relationship between the GP and the patient could perhaps assist the patient in determining which health professional to contact and when.^16^ Increased focus on eye health could help prevent blindness and vision impairment caused by chronic eye diseases.^17^ An important component is incorporating patient knowledge into the treatment strategy to ensure timely health-service use and appropriate care, including discarded patient knowledge, which is often key to understanding the complexities that older adults must navigate when living with chronic diseases.^18^

This study aimed to find out which primary healthcare professionals older members of the public consider to be relevant to contact in relation to their vision and eye health. The focus was on optometrists, POs, and GPs. The findings may aid in organising eye health services for older adults.

## Method

### Design

This study was part of a health intervention aimed at detecting preventable vision loss in general practice.^19^ We used a phenomenological approach with older members of the public aged ≥60 years to gather their experiences with eye health and their views of relevant health professionals.^20^ This allowed us to understand and describe the participants’ attitudes as they expressed them. Focus group interviews^21^ were conducted using a semi-structured interview guide. This strategy allowed the group to have discussions about culturally and morally acceptable attitudes and behaviours. The older members of the public who took part were not included because they had severe vision impairment, rather, the great majority reported good vision function and the ability to perform their daily tasks. Our goal was to spark a discussion about their self-reported vision, future vision expectations, the significance of social relationships, and, most importantly, their imagined expedient health-seeking conduct connected to vision.

### Setting

This study was conducted in Denmark, a social-democratic welfare state, where general practice coordinates health care and performs the majority of diagnostics.^22^ Every citizen is assigned a default general practice. Treatment in the primary and secondary sectors is tax-funded, and no out-of-pocket payments are required. The frequency of annual visits to the GP increases with age. In 2022, half of citizens aged 70–79 years had at least 10 encounters with GPs within a year.^23^ GPs address 90% of all medical cases^22,24^ and serve as an entry point into the healthcare system, as well as a source of subsequent referrals. Nevertheless, few exceptions are found, one of which is that citizens can book an appointment with a PO without a referral from their GP. POs are tax-exempt organisations that operate independently in the primary sector. GPs and POs can both access patient records and refer patients to hospital-based clinics. Optometrists typically operate in privately owned enterprises that are not part of the public healthcare system, which means they cannot refer to or access patient records. Visual acuity tests are often free of charge in optician shops. Optometrists can also perform diagnostic tests, such as fundus photography and tonometry, but they are not permitted to make diagnoses. Before the patient meets the ophthalmologist, POs and hospital-based eye clinics might use optometrists to perform a variety of examinations.

### Data generation

Data were generated in the Copenhagen Capital Region between March and June 2022. Data consisted of four semi-structured focus group interviews with older members of the public,^21^ which were audio-recorded and lasted between 58 and 90 minutes.

The interviews followed an interview guide that covered the following three major themes: 1) the role and significance of one’s vision in everyday life; 2) social relations and identity; and 3) intentional health-seeking behaviours. All interviews began with an explanation of how they were part of a study on general practice detection of vision impairment.

The inclusion criteria were as follows: 1) being aged ≥55 years; and 2) residing in the outskirts of the capital city. A total of 21 participants aged 60–87 years (mean = 72 years) were included after 38 expressed interest in participation (see Table 1). The group composition was guided by an ambition of information power in data.^25^ Participants were recruited through a national non-governmental organisation (NGO) that promotes age-related issues,^26^ and interviews took place in the NGO offices. The participants were separated into the following three interview groups: those with no known eye diseases (*n* = 13); those with cataracts (*n* = 3); and those with other known eye diseases (*n* = 5). The first group were split into two focus groups. The themes in the interview guide were the same in all four interviews but minor changes were made to the questions posed to account for differences in experiences related to health-seeking behaviour.

**Table 1.** Participant characteristics

Sex^a^	Diagnosis	Age, years^b^	Current occupation	Education level^c^
Female	Cataracts	72	Retired	NA
Male	Cataracts	71	Retired	Short
Male	Cataracts	70	Retired	Long
Female	No known eye disease	66	Retired	Short
Male	No known eye disease	72	Retired	Medium
Female	No known eye disease	70	Retired	NA
Male	No known eye disease	75	Retired	NA
Female	No known eye disease	67	Retired	NA
Female	No known eye disease	80	Retired	Short
Female	No known eye disease	80	Retired	Medium
Female	No known eye disease	60	NA	NA
Female	No known eye disease	74	Retired	Medium
Female	No known eye disease	67	Retired	Long
Female	No known eye disease	73	Retired	Short
Female	No known eye disease	71	Retired	Short
Female	No known eye disease	NA	Retired	Short
Female	Other known eye disease	87	Retired	Short
Female	Other known eye disease	75	Retired	Short
Female	Other known eye disease	78	Retired	Medium
Female	Other known eye disease	77	Retired	Medium
Female	Other known eye disease	66	Retired	Medium

^a^Female, *n* = 17; male, *n* = 4. All of Danish ethnic descent. ^b^One participant did not give their age. ^c^Short = 2–2.5 years; medium = 3.5–4 years; and long = ≥5 years. NA = not available.

Given the broad inclusion criteria and differences in diagnostic status across the participant group, the participants were asked their own perception of their vision using the Visual Function Questionnaire-25 (see Supplementary Table S1).^27^

### Data analysis

Focus groups were audio-recorded and transcribed verbatim. Interviews were analysed using thematic analysis.^28^ In the first round, all transcripts were read and coded manually and openly, after which interviews were thematised separately. The authors integrated key themes from the interviews to learn about participants’ perceptions of health professionals’ responsibility for age-related eye health. Following conversations, pertinent themes were defined, and transcripts were coded in NVivo (version 1.7), appropriately. The findings are reported using the Standards for Reporting Qualitative Research.^29^

### Data ethics

The study was carried out following the Helsinki Declaration^30^ and the General Data Protection Regulation. All participants signed a written informed consent form. Participants are kept anonymous, and no specific geographical locations are used.

## Results

**Figure 1.**
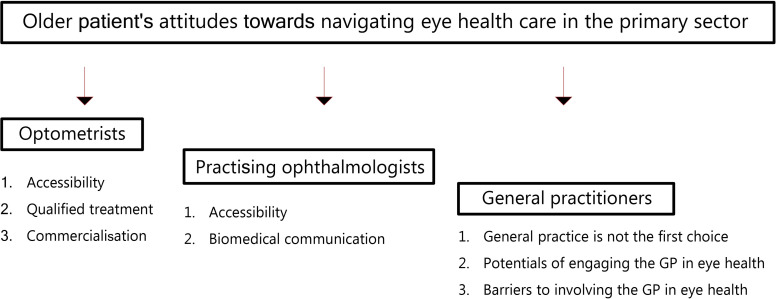
Analytical themes

We present our findings on older members of the public's attitudes towards optometrists and POs being the most relevant health professionals to contact in the following section. We then explain how participants discovered several possibilities for strengthening the role of general practice, and how these possibilities are currently unexplored. Figure 1 depicts an overview of the analytical themes.

### Perceptions of optometrists

Participants described some degree of vision impairment was as a normal part of the ageing process. When experiencing visual changes, the majority of the participants saw optometrists as the ‘initial contact’. Overall, optometrists and participant’s relationships with them were described positively. The following three themes were generated: 1) accessibility was specifically noted as a factor in deciding which health provider to contact first; 2) optometrists were perceived as highly qualified and customer-focused; however, as optometrists commercialise eye health, 3) the relationship was viewed by some participants as difficult.

#### Accessibility

Optometrists were perceived as highly accessible, owing to extended hours, including Saturdays, and the ability to walk in from the street and book an appointment. The participants were asked about whom to contact first when experiencing vision changes in the following excerpt:

*'Well, I would call an optometrist.* […] *it could take maybe six months before I could see a PO. So I’d go see an optometrist and find out if the optometrist said "You need to see a PO".'* (Female, aged 80 years, no known eye disease)

Here the participant perceived the optometrist as the entrance point to the healthcare system. The optometrist is able to perform an eye examination and encourage the older person to contact a PO if relevant, based mainly on accessibility.

#### Qualified treatment

All of the participants had their vision checked by an optometrist at some point. Optometrists were viewed favourably. They were described as service-oriented and proficient in general. Participants expressed considerable confidence in optometrists’ judgements and highlighted their abilities to determine whether additional examinations by a PO were required:

*'He* [the optometrist] *could give you a clue so you can tell the PO "I saw the optometrist who told me I have first signs of glaucoma". Then the PO will see you immediately.'* (Female, aged 67 years, no known eye disease)

The optometrist is here identified as a gatekeeper who could identify what further treatment may be necessary. More importantly, they were viewed as someone who could provide them with the necessary keywords to bypass the long waiting list at POs and instead secure an acute consultation for the older person.

#### Commercialisation

In addition to accepting the optometrists’ judgements and commending them for their qualifications, participants problematised their relationship with the optometrists to the point where they became customers rather than patients:

*'If you go see an optometrist, well that’s a service trade where you’re a customer* ... *'* (Female, aged 74 years, no known eye disease)

The participants were aware of the commercialisation of optometry shops and felt compelled to be critical. However, the discussions on the commercialisation of optometrists were often followed by stories in which participants were advised to not spend money on new glasses but to see a PO before having new glasses made:

*'*… *an optometrist needs to make money, but on the other hand, I have to admit that I’ve been to the optometrist where they told me that it did not prove necessary to buy new glasses yet* […] *so I guess that’s fair, right? But it’s just that thought that it’s pure business.'* (Female, aged 66 years, no known eye disease)

Thus, the experiences were positive, but the concern regarding whether the optometrist was focused on their profit rather than on the older person's health needs underscored the relationship.

### Perceptions of POs

Next to optometrists, participants perceived POs as relevant to be knowledgeable about vision and eye health. Participants who had initially contacted a PO based their decision on a family history of eye diseases; they were aware that a consultation with a PO could be booked and expressed a desire for the most qualified care. Participants identified the following two themes as major barriers to the PO: 1) restricted accessibility; and 2) biomedical communication.

#### Accessibility

The PO was regarded to be difficult to reach. The study’s setting in the Copenhagen Capital Region provided optimal access to specialist doctors and POs placed within a fair geographical distance of the patient, hence accessibility was not connected to geography. Rather, the accessibility issue in this data is related to difficulty in establishing contact and booking consultations. POs were difficult to reach by phone, and some participants had difficulty booking consultations using online platforms. Waiting lists of >6 months were cited as making it very difficult to have your eyes examined by a PO. Overall, it took some effort to book a consultation with the PO, and the participants expressed frustrations with the extended wait:

*'You have to book the next consultation immediately* [after your visit]*.'* (Male, aged 71 years, cataracts)

*'*… *I can’t book a consultation a year in advance. I can only book six months in advance.* […] *So it’s in my diary: call and book a consultation with the PO.'* (Female, aged 72 years, cataracts)

#### Biomedical communication

POs were recognised as experts in performing eye examinations and were described as highly competent. Their medical capabilities were not called into question. What participants questioned was their ability to work with a person-centred approach, which was understood as providing care that is responsive to the individual's life circumstances, including other health-related conditions that the patient may encounter. Thus, addressing the entire person rather than just the eyes. Several times the level of communication was noted as being low and insufficient, as exemplified here:

*'So, I choose the PO, because that should basically be where expertise is found. I mean, they are the ones who are medically trained. But it’s funny, because at the same* [time they] *might not be the most communicating members of the medical world. I mean, I’m sure they’re very skilled at looking into the eyes and performing diagnostics, but they are not particularly talkative or empathetic. They’re like, very specific.'* (Female, aged 71 years, no known eye disease)

The participants reported a lack of patient support and knowledge about their condition(s). The PO identified and expressed their diagnosis, but what this meant for the patient — both in terms of daily life management and disease progression in general — was not felt to be adequately covered. This was the case when a participant shared her experience with a glaucoma diagnosis:

*'Well he* [PO] *was not a man of many words, so he says "you’ve got glaucoma". Okay, and you just sit there.'* (Female, aged 87 years, other known eye disease)

### Perceptions of GPs

The participants did not perceive their GP to be a relevant health practitioner to consult with eye health unless the situation was acute. However, the GP was identified as an important figure in treating patients’ overall health conditions, including age-related conditions and function loss. We will elaborate on the role of the GP by addressing the following generated themes: 1) general practice is not the first choice; 2) the potentials of engaging the GP in eye health; and 3) barriers relating to involving the GP in eye health.

#### General practice is not the first choice

The interview guide asked explicitly which health professional was immediately appropriate to contact when experiencing a vision change, while the three questions that followed asked about the GP’s current or desired role in eye health. Participants had little experience with contacting a GP about eye health; instead, many hypothetical discussions about when to consult a GP were initiated:

*'Well, for instance, something like an allergy. I mean, the first time you catch something like that you don’t know if it’s one or the other thing going on. And then you can’t stand it — that the eyes are itching or streaming and such. But other than that, I don’t think the GP is particularly interested.'* (Female, aged 71 years, no known eye disease)

The participant here mentioned allergy symptoms as factors impacting eye health. However, they concluded by saying that they assumed the GP was only interested in allergy-related eye problems. Their perspective of relevance was thus dual, as they thought that both patients and GPs regarded eye health as unimportant in general practice. This is also expressed in another passage, in which all participants initially said *‘no’* when asked if they had ever visited their GP for something related to their eyes, and one participant concluded by saying: *'I wouldn’t bother him with that.'*

Overall, the participants did not perceive that their eyes and vision were concerns for their GPs, and data lacked stories from participants discussing their vision with their doctors:

*'*… *I wouldn’t start with contacting my GP unless it was painful.'* (Female, aged 67 years, no known eye disease)

#### Potential of engaging the GP in eye health care

The GP-related questions in the interview guide asked participants if they could conceive of ways a GP may participate in monitoring prospective changes in vision, whether their GP asked them about their vision, and if they had any suggestions for how a GP could help them with their eye health. The participants agreed to a large extent that general practice offered potential for eye health care, particularly in detecting early signs of visual impairment, being in charge of further referrals, and managing issues affecting quality of life such as dry eyes. This did not imply that GPs should be in charge of the diagnoses, but as one participant put it:

*'Well, they* [GPs] *can perform a few examinations, but they just need to refer the patient further. Or, "just" is not "just" because you only go see an ophthalmologist if you detect something is wrong, right?'* (Female, aged 72 years, cataracts)

This underlines that patients can only act on symptoms if they can feel or observe them. Participants expressed a need for a more open and holistic dialogue, such as once a year, to discuss vision, as illustrated by the following participant:

*'*… *well just a short test right? It’s not so dramatic when you’re at this level* [primary sector] *and then you can be referred to other specialists. I could actually imagine that would work.'* (Female, age not available, no known eye disease)

In Denmark, GPs can arrange such an annual consultation for those with chronic conditions, and these yearly consultations were popular among the participants in our study. They were also recognised as a possible setting to address vision and overall function loss.

#### Barriers to involving the GP in eye health

The participants all agreed that inefficient structures guide GPs’ work:

*'And I can get so angry because I don’t think it's fair working conditions we offer the GPs.'* (Female, age not available, no known eye disease)

Participants highlighted that consultation time is too short and that it is inconvenient for patients because they must book one consultation per concern and thus feel unable to explain their overall health condition. Even when it came to lengthy sessions, one of the participants stated:

*'*… *you don’t get to talk about everything, whether that’s eyes or not eyes. Generally, you’re left with stuff that you could worry about. There’s not always time to discuss it.'* (Female, aged 72 years, cataracts)

Participants expressed concerns about GPs’ vision and eye health care competence. Some challenged why a GP should assess one’s vision when optometrists and POs were available:

*'Well, I have to say that the GP is not nearly as good as a PO. He’s actually not nearly as good as an optometrist.'* (Female, aged 80 years, no known eye disease)

The participant showed that they were fully aware of the key skills of POs and optometrists in examining the eyes, which is obviously outside the scope of GPs’ expertise. In this respect, further involvement of the GP should be committed to the key competencies of GPs, which include a patient-centred approach and the ability to incorporate patient needs into the treatment plan.

## Discussion

### Summary

This study found that older members of the public considered optometrists and POs to be the most obvious health experts to consult about vision and eye health. When asked directly, the GP was also identified as relevant. As a result, the participants sought treatment from health professionals who were directly qualified for the symptoms they were experiencing or expect to face in the near future as part of ageing. However, participants perceived the GPs’ role in vision and eye health care as unclear, and the current denigration of general practice risks missing potential benefits of robust engagement with general practice. This involves a focus on patients' overall health and function, as well as any comorbidities influencing care trajectories.

### Strengths and limitations

Focus group discussions provided the opportunity to listen in on older members of the public's conversations about primary healthcare providers and their responsibility for eye health and vision. To engage all participants special emphasis was placed on facilitating conversations around the table. Thus, participants’ opinions mirrored comments that are considered socially acceptable or common sense.^21^ This is seen as a strength in our findings since participants’ perceptions were shared in an environment where consensus is sought.

The participants in this study faced minor difficulties in their daily lives as a result of their vision impairment (see Supplementary Table S1). All participants, however, had their vision checked by an optometrist and wore glasses. As a result, their interactions with optometrists were more intense than those with POs. The area of the study setting is characterised by a close geographical proximity to POs, and therefore participants may be more likely to seek specialised doctors than those in more rural areas. The ideal access to specialised doctors may introduce bias. Conversely, it may be a strength as the findings are not influenced by geographical access issues. A limitation of this study is the majority of female participants, making it difficult to address gender-specific practices in health-seeking behaviour.^31^

We exclusively interviewed ethnic Danes from the capital area. Existing research on disparities in eye health has identified ethnic minority status as an indicator of vulnerability and low compliance.^32–35^ We are unable to report on this aspect based on our data, but refer to a Danish study that concluded a generally low level of health literacy among patients with retinal diseases.^36^ The homogeneity of the participants is a strength because it supports the information power of our findings.^25^ The findings are not intended to be generalisable to all older members of the public in Denmark, but rather to reveal perspectives from a group that 1) represents a large population group in the older population; 2) on a specific inquiry; and 3) with the goal of uncovering which primary healthcare professionals’ older members of the public perceive as relevant to contact concerning eye health, and gaining an understanding of culturally acceptable health-seeking behaviour.

Diabetic eye diseases are a threat to vision in many countries and are a major focus of eye health research.^37,38^ We did not include an emphasis on diabetic eye diseases because general practice is already involved in treatment and management of diabetes.

### Comparison with existing literature

Although eye health is a minor topic in general practice, our findings support the assertion that the GP may be a significant stakeholder owing to the GPs’ close and regular interaction with patients ensuring timely disease detection.^39^ According to a Canadian study, public knowledge of asymptomatic eye disease is low.^16^ This requires health professionals to address eye health, identify subtle vision changes, and know what questions to ask. According to a survey of UK GPs, general practice is not good at identifying red flags concerning eyes,^40^ and primary care providers are not good at communicating eye-care information to patients.^41^ In this regard, the GP may fail to diagnose asymptomatic eye disease, necessitating referral to the secondary sector.^42,43^ The GP may act as the initial health professional assessing the eye health of the individual and provide referral for necessary cases.^44–47^

Studies on screening programmes predominate in literature on the detection of vision impairment in general practice.^48,49^ Such courses do not always require heavy equipment^50^ and might be carried out by administering a simple questionnaire on the patient’s appraisal of their vision.^44^ However, visual screenings in primary care have shown mixed results; for example, in an intervention reported by Smeeth *et al* they were unable to find improved visual outcomes,^49^ and the US Preventive Services Task Force recently issued a recommendation statement on screening for impaired visual acuity in older adults, finding insufficient evidence to determine the benefits and harms of screening.^51^ Given the risk of overdiagnosis, observations on the potential unintended effects of screening programmes must be examined.^52^ This includes self-screening, which has been linked to anxiety and excessive use of health resources.^53^

### Implications for research and practice

Because of their often asymptomatic nature, eye diseases, such as AMD and glaucoma, can be difficult to identify. Older members of the public's attitudes toward relevant health professionals’ responsibilities are significant because they play a pivotal role in citizens’ health-seeking behaviours and can help more citizens preserve their vision longer. According to this study, optometrists, followed by POs, are identified as relevant health providers. Because optometrists and POs are experts in vision and eye health, the older members of the public included in the study reported an expediently health-seeking behaviour. This study was carried out in a setting with good geographical access to specialists, which may explain participants’ preference for optometrists and POs over general practice. However, the commercial objective of optometrists and the biomedical communication of POs presented challenges to the participants. They expressed a wish for health professionals to apply a focus on them as people — which they found in the service-oriented optometrists — with a non-commercial focus and high knowledge level on their overall health, including eye-health; much like the role of general practice. This study cannot reflect on existing experiences with general practice, but we have highlighted the potential of general practice based on the participants’ statements of increasing focus on older people's overall health and function. GPs are concerned with the patients’ daily life, including multimorbidity and long-term health. This gives GPs a key role as the patient’s first point of contact in the healthcare system, where they may help manage vision impairment and explain diagnoses given by specialists. General practice is in charge of delivering primary health care to all citizens and is frequently in charge of managing chronic conditions such as hypertension and diabetes. In this context, age-related loss of function, including vision, is a critical issue to address owing to its impact on the patient’s ability to self-care, as well as the patient’s physical and mental health. This could be accomplished by asking questions about recurring falls or limits in daily activities, as well as making accurate referrals. In this regard, the findings are relevant to a debate on general practice sustainability, because early detection of vision impairment reduces the burden for patients and can reduce workload in general practice and ophthalmology practice.
